# Effects of nitric oxide synthase inhibitor ω-Nitro-L-Arginine Methyl Ester, on silica-induced inflammatory reaction and apoptosis

**DOI:** 10.1186/1743-8977-3-14

**Published:** 2006-11-07

**Authors:** He Wang, James Leigh

**Affiliations:** 1 Discipline of Public Health, University of Adelaide, 10 Pulteney Street, Adelaide, 5005 SA, Australia; 2 School of Public Health, University of Sydney, Sydney, 2006 NSW, Australia

## Abstract

**Background:**

Although nitric oxide is overproduced by macrophages and neutrophils after exposure to silica, its role in silica-induced inflammatory reaction and apoptosis needs further clarification. In this study, rats were intratracheally instilled with either silica suspension or saline to examine inflammatory reactions and intraperitoneally injected with ω-nitro-L-arginine methyl ester (L-NAME), an inhibitor of nitric oxide synthases, or saline to examine the possible role of nitric oxide production in the reaction.

**Results:**

Results showed that silica instillation induced a strong inflammatory reaction indicated by increased total cell number, number of neutrophils, protein concentration and lactate dehydrogenase (LDH) activity in bronchoalveolar lavage fluid (BALF). There were no significant differences in these indices between silica-instilled groups with and without L-NAME injection (p > 0.05) except LDH level. The results also showed that apoptotic leucocytes were identified in BALF cells of silica-instilled groups whereas no significant difference was found between silica-instilled groups with and without L-NAME injection in the apoptotic reaction (p > 0.05). Silica instillation significantly increased the level of BALF nitrite/nitrate and L-NAME injection reduced this increase.

**Conclusion:**

Intratracheal instillation of silica caused an obvious inflammatory reaction and leucocyte apoptosis, but these reactions were not influenced by intraperitoneal injection of L-NAME and reduced production of NO. This supports the possibility that silica-induced lung inflammation and BALF cell apoptosis are via NO-independent mechanisms.

## Background

Silica exposure results in an initial inflammatory reaction and subsequent fibrosis. During this process, various agents such as cytokines and free radicals are produced and these agents in turn regulate the development of the inflammation and fibrosis [1]. Nitric oxide (NO), a small molecule with multiple biologic functions, has been shown to be overproduced by alveolar macrophages and neutrophils as well as other cell types after exposure to intratracheal instillation of silica [2-4]. It has also been demonstrated that γ-interferon and TNF-α, which are produced in silica-induced response [5], can induce synthesis of NO [6].

NO is a molecule that can readily pass through the cell membrane and exert its action on cells. It is involved in vessel dilatation, inhibition of platelet aggregation [7] and host defence. It is also an apoptosis inducer for some cell types [8]. Since silica-induced apoptosis of bronchoalveolar leucocytes has already been demonstrated [9] and probably has a role in the evolution of silica-induced inflammation and fibrosis, NO may induce leucocyte apoptosis to regulate these pathological reactions. However, there are also evidences that some apoptotic change occurs via a nitric oxide-independent pathway [10,11]. It is not known whether leucocyte apoptosis in silica-induced inflammation occurs via the nitric oxide-dependent or independent pathway.

Although NO seems to be beneficial by induction of apoptosis in inflammatory cells, the NO produced in a silica-induced lung reaction may be harmful. That is because nitric oxide can combine with superoxide to form peroxynitrite free radical and this substance can cause severe lung damage [2,12,13]. Indeed, other harmful agents such as asbestos fibres [14] and ozone [15], which induce lung damage, have been demonstrated to enhance production of NO in vitro and in vivo although the role of NO in the process is uncertain. Even in the case of peroxynitrite, its exact role in pathological processes is contradictive. Current knowledge indicates that both NO and peroxynitrite may have dual or beneficial and harmful effects in inflammatory reactions depending on situation.

ω-nitro-L-arginine methyl ester (L-NAME) is a NO synthase inhibitor and inhibits the production of NO by inducible NO synthase and constitutive NO synthase. In silica-induced inflammatory reaction, inducible NO synthase gene expression increases in alveolar leucocytes and the production of NO is likely to be via the increased gene expression of NO synthase [3]. In the present study, silica-induced inflammation and apoptosis were quantified in intratracheally (it) silica-instilled rats with and without L-NAME injection to see if this compound could inhibit the inflammatory reaction and apoptosis.

## Results

### Inflammatory reaction

**Figure 1:**
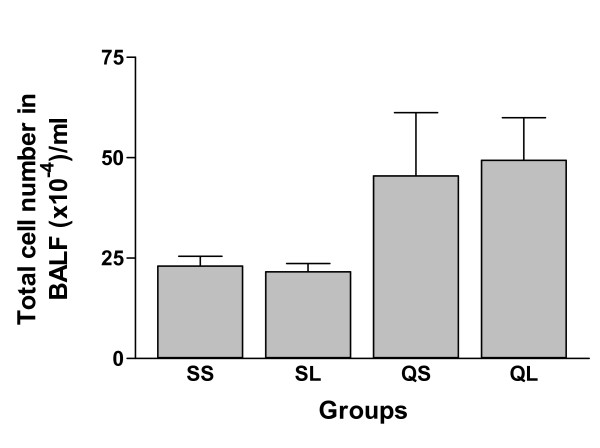
Total cell number in bronchoalveolar lavage fluid of rats after intratracheal instillation of silica or saline and intraperitoneal injection of saline or ω-nitro-L-arginine methyl ester (L-NAME). QL = silica (quartz) + L-NAME; QS = silica + saline; SL = saline + L-NAME; SS = saline + saline (These abbreviations also apply to subsequent figures) (mean + se).

Intratracheal instillation of silica induced an obvious inflammatory reaction in silica-instilled rats but not in the saline-instilled rats. Intraperitoneal injection (ip) of L-NAME in a dosage of 15 mg/kg/day did not influence the inflammatory reaction significantly. There was no significant difference in total cell number of bronchoalveolar lavage fluid (BALF) between (it) silica + (ip) saline and (it) silica + (ip) L-NAME groups (p > 0.05, Figure 1). Both silica-instilled groups showed larger numbers of total cells than the saline-instilled groups but the difference was not statistically significant possibly due to the large individual variation in silica-instilled groups.

**Figure 2:**
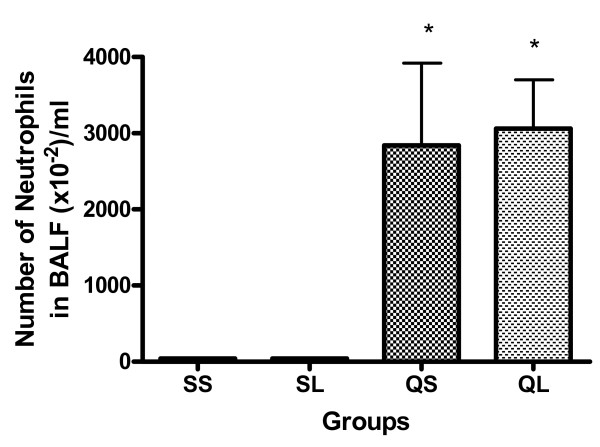
Percentage of neutrophils in BALF cells of rats after intratracheal instillation of silica or saline and intraperitoneal injection of saline or ω-nitro-L-arginine methyl ester (L-NAME) (mean + se). * There is a statistically significant difference in comparison with control groups.

The number of neutrophils in BALF increased markedly in both of the silica-instilled groups compared with saline-instilled rats (Figure 2). There were no significant differences between saline- and L-NAME-treated groups in either silica-instilled or saline-instilled rats.

**Figure 3:**
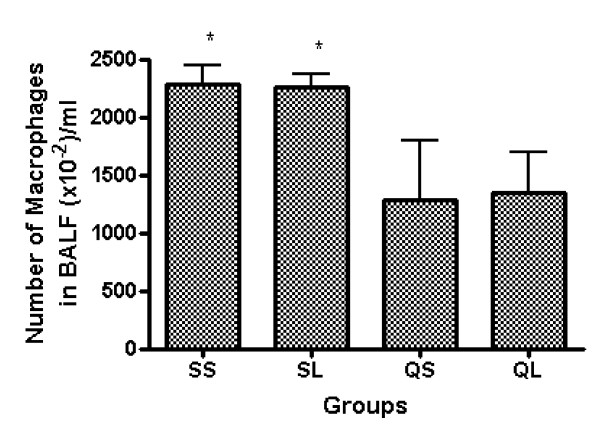
Percentage of macrophages in BALF cells of rats after intratracheal instillation of silica or saline and intraperitoneal injection of saline or ω-nitro-L-arginine methyl ester (L-NAME) (mean + se). * There is a statistically significant difference in comparison with control groups.

The number of macrophages in silica-instilled rats decreased significantly compared with saline-instilled groups (p < 0.01). This might be a reflection of increased death of alveolar macrophages because of silica toxicity. L-NAME had no influence on the number of macrophages in both silica- and saline-instilled groups (Figure 3).

**Figure 4:**
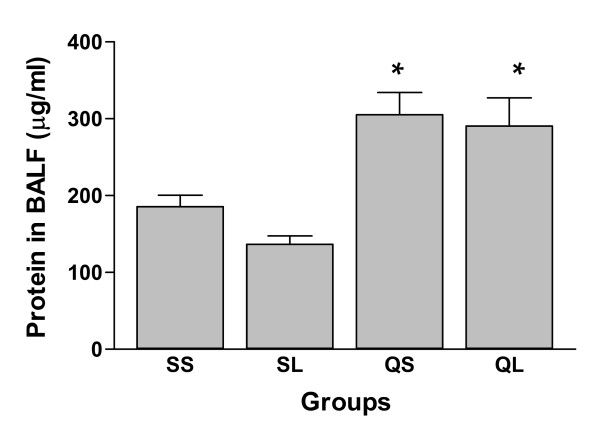
Protein concentration in bronchoalveolar lavage fluid of rats after intratracheal instillation of silica or saline and intraperitoneal injection of saline or ω-nitro-L-arginine methyl ester (L-NAME) (mean + se). * There is a statistically significant difference in comparison with control groups.

The protein concentration in BALF of silica-instilled groups significantly (p < 0.05) increased compared with saline-instilled groups but no significant difference could be detected between the silica-instilled groups with and without L-NAME injection (Figure 4).

**Figure 5:**
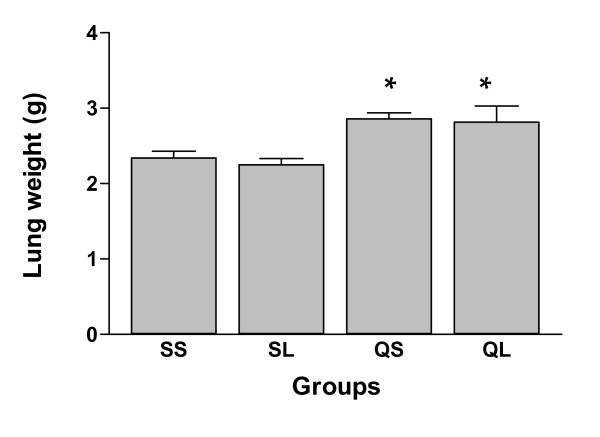
Lung weight of rats after intratracheal instillation of silica or saline and intraperitoneal injection of saline or ω-nitro-L-arginine methyl ester (L-NAME) (mean + se). * There is a statistically significant difference in comparison with control groups.

The lung weight of silica-instilled groups was significantly (p < 0.05) higher than that of saline-instilled groups (Figure 5), but no significant difference was found between the L-NAME and non-L-NAME silica-instilled groups (p > 0.05).

**Figure 6:**
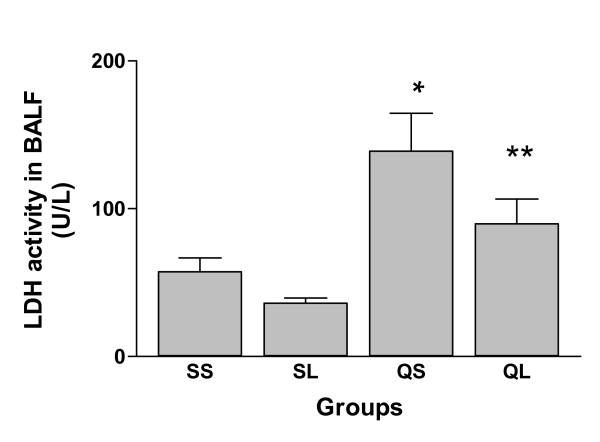
LDH activity in bronchoalveolar lavage fluid of rats after intratracheal instillation of silica or saline and intraperitoneal injection of saline or ω-nitro-L-arginine methyl ester (L-NAME) (mean + se). * There is a statistically significant difference in comparison with control groups. * * There is a statistically significant difference in comparison with saline-injected group.

The lactate dehydrogenase (LDH) activity in BALF from silica-instilled groups was significantly (p < 0.05) higher than that of saline-instilled groups and L-NAME injection reduced the increase of LDH activity induced by silica instillation from 140 U/L to 95 U/L (p < 0.05, Figure 6).

### Apoptotic Reaction

**Figure 7:**
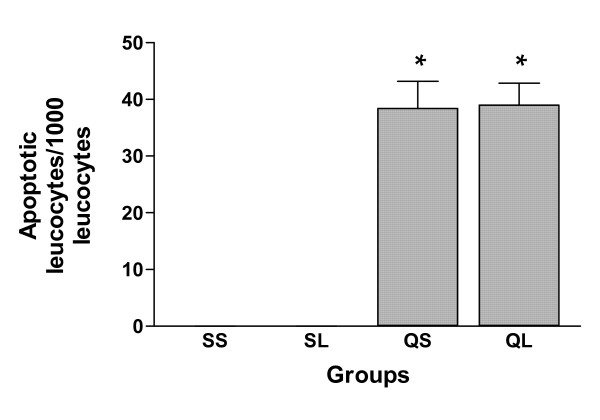
Proportion of apoptotic leucocytes in BALF leucocytes of rats after intratracheal instillation of silica or saline and intraperitoneal injection of saline or ω-nitro-L-arginine methyl ester (L-NAME) (mean + se). * There is a statistically significant difference in comparison with control groups.

**Figure 8:**
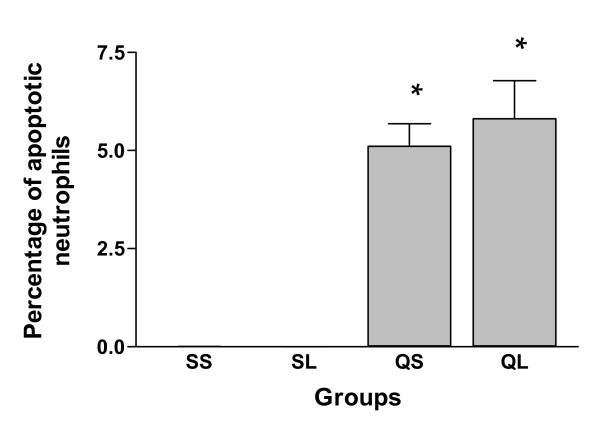
Percentage of apoptotic neutrophils in BALF neutrophils of rats after intratracheal instillation of silica or saline and intraperitoneal injection of saline or ω-nitro-L-arginine methyl ester (L-NAME) (mean + se). * There is a statistically significant difference in comparison with control groups.

**Figure 9:**
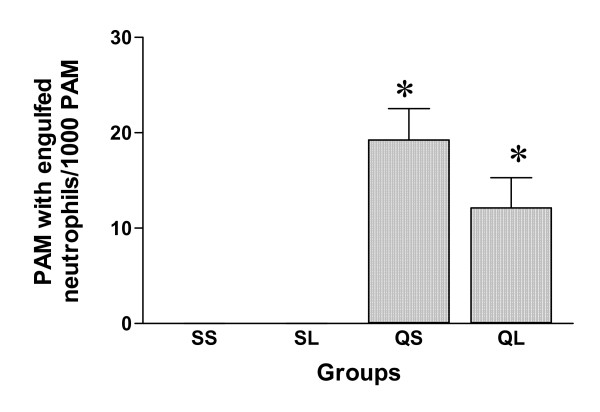
Proportion of pulmonary alveolar macrophages with engulfed apoptotic neutrophils in BALF macrophages of rats after intratracheal instillation of silica or saline and intraperitoneal injection of saline or ω-nitro-L-arginine methyl ester (L-NAME) (mean + se). * There is a statistically significant difference in comparison with control groups.

A number of apoptotic leucocytes were identified in BALF cells of both silica-instilled groups. The apoptotic cells were mainly neutrophils by morphology. Engulfment of apoptotic cells by macrophages was also identified. Comparison of the proportion of apoptotic leucocytes (Figure 7), the percentage of apoptotic neutrophils (Figure 8) and the proportion of macrophages with engulfed apoptotic cells (Figure 9) between the two silica-instilled groups, indicated that the differences were not significant (p > 0.05). In saline-instilled groups, apoptotic cells were extremely few.

### Nitrite/nitrate level in BALF

**Figure 10:**
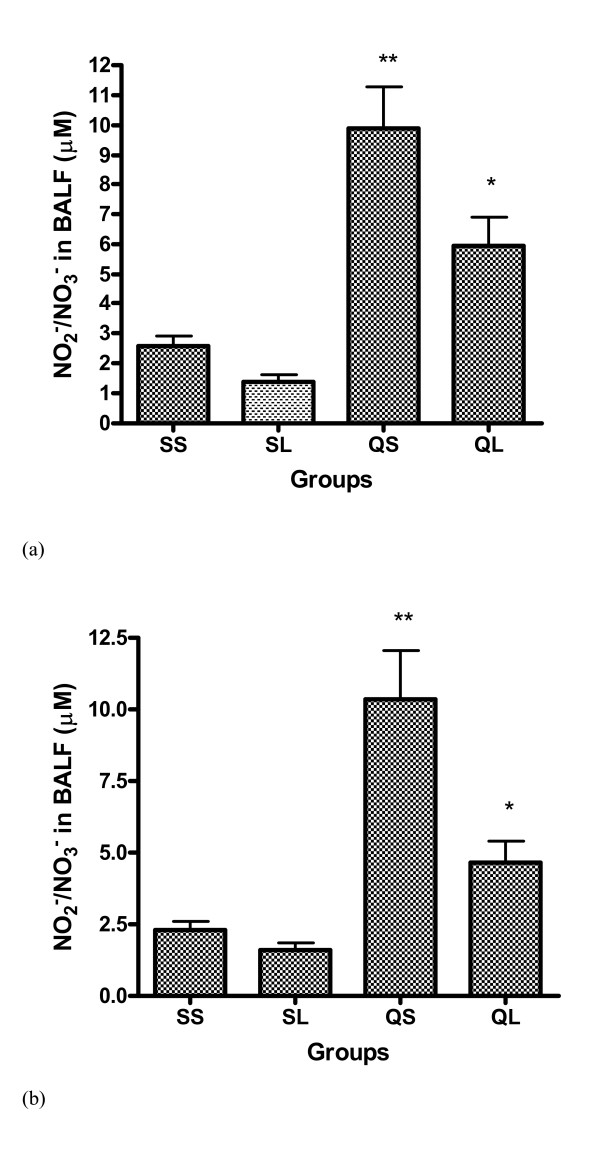
(a) Concentration of NO_2_^-^/NO_3_^- ^(μ*M*) in BALF of rats 2 weeks after intratracheal instillation of silica or saline and intraperitoneal injection of saline or ω-nitro-L-arginine methyl ester (L-NAME) (mean + se). ** There is a statistically significant difference in comparison with control groups. * There is a statistically significant difference in comparison with instillation of silica and injection of saline group. (b) Concentration of NO_2_^-^/NO_3_^- ^(μ*M*) in BALF of rats 4 weeks after intratracheal instillation of silica or saline and intraperitoneal injection of saline or ω-nitro-L-arginine methyl ester (L-NAME) (mean + se). ** There is a statistically significant difference in comparison with control group. * There is a statistically significant difference in comparison with instillation of silica and injection of saline group.

Nitrite/nitrate can be detected in BALF from all the experimental animals. Silica instillation increased the level of nitrite/nitrate in BALF significantly two and four weeks after instillation and L-NAME injection reduced the increase by about 40% at two weeks time point and 55% at four weeks time point (Figure 10).

## Discussion

In this study, a strong inflammatory reaction was induced following silica instillation. At the administered dosage, L-NAME failed to influence the silica-induced inflammation indices. The L-NAME dosage was above the effective dosage in similar studies for the inhibition of lung injury [16-19] and actually reduced the level of nitrite/nitrate in BALF from silica-instilled rats in this experiment.

The results in this study may be an indication that although nitric oxide may be an important marker in the development of lung injury, it does not play a pivotal role in silica-induced inflammation under the conditions of this experiment. In a previous study with a rat model [20], intratracheal instillation of silica (100 mg/kg BW)caused overproduction of both superoxide and nitric oxide 24 hours later. Nitric oxide has been demonstrated to be able to combine with superoxide to form peroxynitrite which is a much stronger harmful agent to lung tissue [21,22]. However, NO synthase inhibitor L-NAME did not reduce lung inflammatory reactions in the present study although the level of nitrite/nitrate in BALF from silica-instilled rats was reduced by the drug. L-NAME had also been shown to decrease NO as well as peroxynitrite production in silica-induced acute lung injury [20] in a previous study. The damaging role of NO or peroxynitrite in silica-induced lung injury could not be verified by injection of L-NAME which has been widely used as a NO synthase inhibitor. The dependency of silica-induced lung inflammation on NO or peroxynitrite is open to question.

NO has been demonstrated to cause apoptosis [23]. Induction of inducible NO synthase by exogenous agent can also lead to cell death by apoptosis [24]. Macrophages cause apoptosis of other cell types through the release of nitric oxide [25]. In silica-induced inflammation, increase in inducible nitric oxide synthase activity, nitric oxide overproduction and leucocyte apoptosis have all been demonstrated [26,27]. This suggests that there is a probability that the silica-induced nitric oxide might induce neutrophil apoptosis to regulate the evolution of the silica-induced inflammation. However, in this experiment, L-NAME did not influence leucocyte apoptosis significantly. Since the dosage is well above effective and actually reduced the BALF level of nitrite/nitrate, it is possible that nitric oxide plays only a minor role in the leucocyte apoptosis of silica-induced inflammation.

It is noted that chemicals can cause cell apoptosis by either nitric oxide-dependent or nitric oxide-independent mechanisms [28]. For example, bisphosphonates induce apoptosis in mouse macrophage-like cells *in vitro *by an nitric oxide-independent pathway [29]. These workers demonstrated that the apoptosis induced by bisphosphonates was not due to increased synthesis of NO and could not be prevented by inhibitors of NO synthase. NO also failed to induce apoptosis in oligodendrocytes [30].

Peroxynitrite can be produced in silica-induced lung injury and this endogenous substance can induce either necrosis [31,32] or apoptosis[33-36]. In this experiment, L-NAME did not influence either leucocyte apoptosis or lung inflammation significantly. Since peroxynitrite might be overproduced in silica-induced inflammation of this study and L-NAME actually reduced the overproduction of NO, there is a possibility that leucocyte apoptosis in silica-induced inflammation was via a peroxynitrite-independent pathway.

It is interesting that L-NAME at an effective dosage, failed to influence silica-induced inflammation as well as leucocyte apoptosis. This supports the hypothesis that the regulation of apoptosis might influence the evolution of silica-induced inflammation. For example, in a previous study, it was demonstrated that enhancement of apoptosis by IL-10 accelerated the resolution of acute inflammation induced by LPS [37]. It was noted that L-NAME inhibited silica-induced LDH activity increase in BALF in this experiment. This inhibition may also be related to a NO-independent pathway since it has been demonstrated that L-NAME can reduce LDH release by cultured cells independent of NO reduction [38,39].

In regard to the role of NO in injuries caused by various invading agents, conflicting results have been presented. A number of experiments showed that NO was harmful to the exposed cells or tissues [2] whereas some experiments indicated that NO actually played a protective role to the exposed tissues or cells [40,41]. Moreover, in an in vitro study NO was demonstrated to play a dichotomous role in asbestos induced harmful effects [42]. The result of this study was the outcome of four weeks after one off exposure of silica and continuing administration of L-NAME. Although this study did not directly measure the activity of NO synthases, the production of NO estimated by nitrite/nitrate level in BALF was dramatically reduced by the injection of L-NAME. Since it has been reviewed that direct link between silica-induced NO production and pathogenesis was obtained using iNOS knockout mice, and iNOS knockout provided protection in sub-chronic exposure of silica but not in acute exposure [43], the complexity of NO synthases in silica-induced inflammation is shown. Further studies are needed to reveal the exact role of NO in silica-induced harmful effects.

## Conclusion

This study demonstrated that L-NAME, a nitric oxide synthase inhibitor, did not influence the inflammatory reaction and apoptosis of neutrophils after exposure to intratracheal silica in rats at the dosage of 15 mg/kg/day for four weeks. Because this dosage was more than10 times of L-NAME ED 50 and it actually reduced the production of NO in silica-instilled lungs, NO probably played little role in the development of inflammation and leucocyte apoptosis in silica-induced harmful effects. The result is inconsistent with a number of previous studies which support the concept that NO can harm lung tissue in silica-induced effects by combining with superoxide to form peroxynitrite. We conclude that silica-induced harmful effects are probably caused by NO or peroxynitrite independent pathways in the rat model established by intratracheal instillation.

## Methods

### Experimental animals and group

The animals used in the experiments were male Wistar rats. They were purchased from the animal breeding facility of The University of New South Wales (Little Bay, NSW) and held in presterilised cages in the animal holding facility of the National Institute of Occupational Health and Safety. Sterilised food and water were supplied *ad libitum*. Experimental protocols involving rats were in accordance with the University of Sydney regulations and approved by The Animal Care Ethics Committee (ACEC) of The University of Sydney (ACEC number: WOR/7-95/2/2182).

In this study, body weights of the rats at the start of the experiment were 224–235 grams. The rats were grouped by randomised block design into four groups (each of 5): (1) it saline + ip saline; (2) it saline + ip L-NAME; (3) it silica + ip saline; (4) it silica + ip L-NAME.

### Preparation of dust suspensions

Min-U-Sil 5 silica was obtained from Silica Corp, Berkeley Springs, West Virginia (particle size range was 0.6–8.0 μm, 98% < 5 μm diameter; purity was 99.5% α-quartz by X-ray diffraction). A 12.5 mg amount of dust was suspended in 0.5 ml saline or 0.5 ml saline alone, sterilised by autoclave and vortexed before injection. The preparation was free of endotoxin.

### Intratracheal instillation and intraperitoneal injection

Rats were anesthetised by ip injection of a mixture of ketamine (100 mg/kg) and xylazine (3.3 mg/kg, Sigma Chemical Company, St Louis, MO, USA). Before use, the chemicals were dissolved in saline and sterilised by filtration. Intratracheal instillation was performed by a procedure of tracheal exposure. After shaving, the skin in the ventral aspect of the neck was incised in the midline. The trachea was exposed through blunt dissection. Using a 1 ml disposable syringe with a #26 needle, 12.5 mg silica dust dissolved in 0.5 ml of saline, or saline alone, was injected. The incision was sutured with interrupted silk sutures immediately after the injection and the rat was kept in a 30°C incubator until it regained consciousness. Immediately after the recovery of the instilled rats from anasthesia, saline or L-NAME dissolved in saline was ip injected at a dosage of 15 mg/kg. Thereafter, the injection was conducted daily at the same dosage for 4 weeks.

### Bronchoalveolar lavage

Randomly-selected rats were anesthetised with an ip injection of 75 mg/kg of pentobarbital when 4 weeks observation period completed. Laparotomy was performed and the abdominal aorta was exposed. The rats were killed by transection of the aorta. There was no significant difference in body weight among the different groups of rats in each experiment when sacrificed.

After sacrifice, an incision was made into the neck region and the trachea was exposed by trimming of surrounding tissue. A piece of suturing thread was passed under the exposed trachea. A small opening was made by cutting in a lengthwise direction just below the larynx and in transverse direction just below and connected to the lengthwise incision. An 18-gauge needle in plastic tubing was placed into the opening and pushed through the trachea towards the lung. The needle was secured in place with the thread by ligation. A 5 ml volume of phosphate buffered saline (PBS) was injected into the needle with a 5 ml syringe for lavage. The injection process was finished within one minute and the fluid was kept in the lung for an additional minute. The withdrawal process was also finished in one minute. In total, two aliquots of 5 ml PBS were used for the lavage of each rat. The recovered fluid of each rat was pooled and the volume recorded. The recovered volume of BALF was between 9.0 to 9.7 ml and no significant difference could be detected among the different groups of rats in the recovered volume.

### Counting of BALF cells

The total cell number was counted with a haemocytometer. A 50 μl volume of obtained lavage fluid was transferred to a plastic vial and mixed with the same amount of trypan blue solution (Sigma Chemical Company, St Louis, MO, USA). The well-mixed fluid then was transferred to a haemocytometer and the BALF cells were counted under a microscope.

### Slide preparation, staining and differential counting

A 100 μl volume of the BALF was placed on a slide by Cytospin centrifugation immediately after the lavage (600 rpm, 5 min, Shandon, USA). At least two slides were prepared from each rat and stained with Diff-Quik (Lab. Aids Pty. Ltd., Narrabeen, Australia). The interval between sacrifice of rat to placement of BALF on slides was approximately 15 min.

The slides were read under oil immersion (× 1000). Five hundred leucocytes were counted to determine the frequency of different types of cells by their morphology.

### Measurement of BALF protein

The supernatant of centrifuged BALF was placed in 400 μl polyethylene tubes, in duplicate. The protein concentration was measured by Lowry's method [44] using bovine serum albumin as a standard. The measurement was conducted 1 day after lavage.

### Measurement of lactate dehydrogenase

BALF was centrifuged (3000 rpm, 15 min) and the supernatant was used for measurement of LDH activity. The measurement was performed with a CentrifiChem System 400 using a kit method (Trace Scientific Pty. Ltd., Sydney, Australia) and determined within 3 days after lavage. LDH measurements were performed within 3 days after lavage. The samples were kept in ice during operation and stored in -20°C freezer.

### Identification of apoptosis

The slides were blind-coded before the scoring of apoptotic leucocytes. A minimum of 1000 leucocytes were counted for the occurrence of cells with apoptotic features. A minimum of 500 neutrophils were counted to determine the percentage of apoptotic neutrophils. Apoptotic features included formation of condensed chromatin bodies with sharp edges and convolution of the cell surface [45,46]. Apoptotic macrophages and neutrophils were differentiated by morphology. Apoptotic macrophages were generally larger in size, with clear and non-ciliated cell borders. The key point in discriminating apoptotic macrophages from apoptotic neutrophils is the cytoplasm staining with Diff-Quik. The cytoplasm of apoptotic macrophages is a blue colour, similar to the non-apoptotic macrophages. The cytoplasm of apoptotic neutrophils is light yellow, similar to non-apoptotic neutrophils. Morphological apoptosis of the cells in BALF was confirmed by agarose gel electrophoresis of extracted genomic DNA. In the electrophoresis, cells in BALF with morphological apoptosis showed a ladder pattern, whereas cells without morphological apoptosis lacked such an eletrophoretic feature.

### Scoring of macrophages with engulfed apoptotic cells

A total of 1000 macrophages were counted for the incidence of macrophages with engulfed cells or apoptotic bodies. The proportion of macrophages with engulfed cells or bodies in 1000 counted macrophages was calculated.

### Effect of L-NAME injection on production of NO metabolites

To examine the effects of L-NAME administration on production of NO in silica-instilled lungs, forty male Wistar rats were used to obtain BALF for the measurement of nitrite/nitrate levels. Body weights of these rats at the start of the experiment were 209–220 grams. The rats were grouped by randomised block design into four groups for intratracheal instillation and ip injection (each of 10): (1) it saline + ip saline; (2) it saline + ip L-NAME; (3) it silica + ip saline; (4) it silica + ip L-NAME. At two weeks after instillation, 5 rats in each group were randomly selected and killed for lavage. At four weeks after instillation, all the remaining rats were killed and lavaged. The lavage fluid was used for the measurement of nitrite/nitrate level within two hours. The procedures in instillation, injection, killing and lavage were exactly the same as described above.

Nitrite/nitrate levels in the obtained BALF were measured by a Total Nitric Oxide and Nitrate/Nitrite Parameter Assay Kit (R&D System, Minneapolis MN, USA) with conversion of nitrate to nitrite by nitrate reductase. Absorbances were measured at 540 nm with ELISA microplate reader (Titertek Multiskan MCC/340).

### Statistical analysis

The results are expressed as means ± standard error (se). Student-Newman-Keul's test was used for multiple comparison of different animal groups of each index with the software package Instat because this test method is suitable for multiple comparison. Analyses of variance were also conducted by this software. Statistical significance was preset at p < 0.05.

## Competing Interests

The author(s) declare that they have no competing interests.

## Authors' contributions

HW designed the study, conducted all the experiments and drafted the manuscript.

JL participated in the design of the study, contributed to the discussion and provided funding for the project.

All authors read and approved the final manuscript.
